# Laparoscopic Bowel Injuries Among Gynecologic Patients

**DOI:** 10.1155/DTE.3.241

**Published:** 1997

**Authors:** Francisco A. R. Garcia, Joel M. Childers

**Affiliations:** 1 Department of Obstetrics and Gynecology Johns Hopkins Bayview Medical Center Baltimore Maryland USA; 2 Division of Gynecologic Oncology Department of Obstetrics and Gynecology The University of Arizona Health Sciences Center 2625 North Craycroft, Suite 201 Tucson Arizona 85712 USA

## Abstract

Bowel injury is an uncommon but recognized risk of operative laparoscopy. Because of the significant morbidity that can occur with this complication, it is important that clinicians be aware of its incidence, presentation, and management. This manuscript outlines the common causes of bowel injury, including herniation and traumatic bowel perforation. Management of laparoscopic bowel injuries is discussed and recommendations are made for avoidance of such complications.


*Diagnostic and Therapeutic Endoscopy*, 1997, Vol. 3, pp. 241–247
Reprints available directly from the publisher
Photocopying permitted by license only

© 1997 OPA (Overseas Publishers Association)
Amsterdam B.V. Published in The Netherlands
by Harwood Academic Publishers
Printed in Singapore

### Laparoscopic Bowel Injuries Among Gynecologic Patients: A Critical Review

FRANCISCO, A.R. GARCIA[1] and JOEL M. CHILDERS[2]*

[1]*Department of Obstetrics and Gynecology, Johns Hopkins Bayview Medical Center, Baltimore, Maryland, USA*
[2]*Division of Gynecologic Oncology, Department of Obstetrics and Gynecology, The University of Arizona Health Sciences Center, Tucson, Arizona, USA*

*(Received 26 August 1996; in final form 27 September 1996)*

**Bowel injury is an uncommon but recognized risk of operative laparoscopy. Because of the significant morbidity that can occur with this complication, it is important that clinicians be aware of its incidence, presentation, and management. This manuscript outlines the common causes of bowel injury, including herniation and traumatic bowel perforation. Management of laparoscopic bowel injuries is discussed and recommendations are made for avoidance of such complications.**

*Keywords:* Bowel injury, bowel perforation, herniation, laparoscopic complication

### INTRODUCTION

Bowel injury is an uncommon but recognized risk of operative laparoscopy [1]. This operative complication can result in significant morbidity and even death if unrecognized at surgery. Despite the relative importance of this complication, data is minimal regarding its incidence, clinical presentation and management. Estimating the incidence of bowel injury during modern operative laparoscopy is difficult. Reports attempting to estimate this figure are limited in important ways. The majority of the existing literature reflects the experience during the preoperative era of laparoscopy, predominantly tubal occlusions and diagnostic procedures performed primarily on low risk populations. Furthermore the majority of existing literature on laparoscopic bowel injuries are case reports, small case series and operator surveys which inadequately estimate the incidence of this complication. With the explosion of laparoscopy in terms of numbers, as well as length and complexity of procedures, more frequent and severe complications can be expected.

*Correspondence: J.M. Childers, MD, Division of Gynecologic Oncology, Department of Obstetrics and Gynecology, The University of Arizona Health Sciences Center, 2625 North Craycroft, Suite 201, Tucson Arizona 85712, USA .

Laparoscopic bowel injuries can occur at any time during the operative process. First bowel injury can occur while gaining access to the abdomen during placement of the Veress needle or trocar and sleeve, the primary port in particular. Second, injury to the bowel can occur during the operative procedure via sharp, blunt and even thermal trauma. Finally, post operative bowel herniation may occur at trocar sites. The literature over the last 25 years is replete with case reports and series as well as operator surveys. A review of the existing literature provides insight into the incidence, mechanism, presentations, management, and prevention of laparoscopic bowel injuries [1-39].

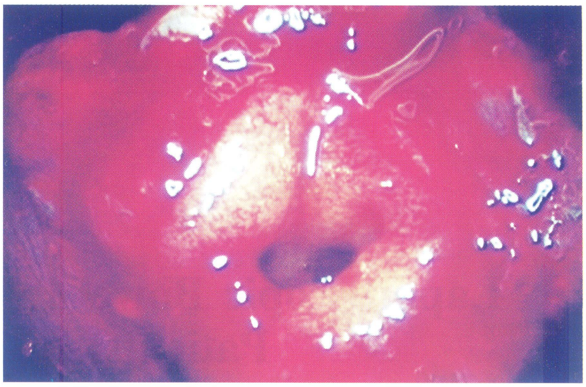

FIGURE 1A Laparoscopic view of a full thickness traumatic small bowel injury.

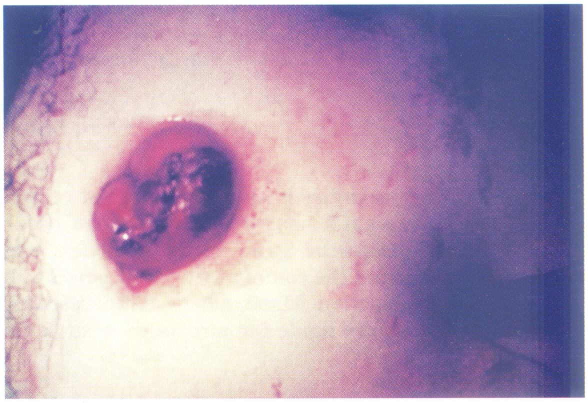

FIGURE 1B The small bowel was exteriorized through the suprapubic port site and repaired with interrupted 3–0 silk suture.

### Bowel Perforation

Traumatic bowel injury is a potentially lethal complication of operative laparoscopy and can occur while gaining access to the abdomen or during the operative process itself. The advent of more advanced operative procedures has led to an increase in the number of bowel injuries occurring with blunt or sharp dissection, as well as thermal energy [17]. Bowel injuries can be further subdivided into those which are recognized at the time of surgery, as opposed to those which escape detection.

Although the true incidence of laparoscopic bowel perforation is unknown, it has been estimated to occur in 1/200 to 1/2000 cases[19–23]. These estimates are based on large surveys and case series of adverse events. In general this data reflects the pre-operative era of laparoscopy with an over representation of diagnostic procedures and tubal sterilizations. Thus these figures undoubtedly represent conservative estimations of a much more common occurrence.

At surgery, laparoscopic injury to the bowel is a frequently unrecognized complication at the time of the primary operation. In these instances the consequences are potentially disastrous. In over 90% of the reported cases this complication was not recognized at the time of the original operation. The small bowel is most often involved (Fig. 1), with the terminal ileum being the most common site of damage, although there are reports of injury to the colon and rectum [24–26]. With regard to primary trocar injury, neither open laparoscopy technique nor the use of disposable trocars with safety shields eliminates the potential for traumatic injury to the bowel [19,24].

The clinical presentation of patients with unrecognized bowel injury can be non-specific and misleading [24,25,27–30]. Symptoms of nausea, emesis and/or abdominal pain are frequently elicited though not always present. Most patients present symptomatically within three to ten days of surgery, though the reported range spans from 18 hours to 23 days [24,25,27,29,30]. Physical and laboratory examination can be misleading as well, and a normal temperature, and white blood cell count can often be present. Absence of free air on radiographs and the absence of abscess on ultrasound exam do not preclude the diagnosis.

In one report of 66 patients with unrecognized bowel injuries, 59% had a normal white count and 33% were afebrile at the time of presentation[24]. All patients with small bowel injuries, had both normal white blood cell count and temperature. Among the patients with both leukocytosis and fever, all had large bowel trauma. Additionally among patients who were imaged radiologically or with ultrasound, negative findings were present in 66% and 33% of cases, respectively.

Despite the potentially lethal consequences of unrecognized bowel injury, death is a rare complication of laparoscopic bowel damage[23,31]. When it does occur it is usually in the setting of large bowel injury, and associated with abdominal exploration which is delayed beyond 72 hours after the onset of symptoms. Death results from sepsis and its complications, including disseminated intravascular coagulation, shock and adult respiratory distress syndrome. Though much less common, death as a result of small bowel injury has occurred.

### Electrical vs. Mechanical Injury

Electrical burns have been assumed to represent the most common cause of bowel damage. This was based on early experience utilizing monopolar electricity in the setting of laparoscopic sterilization [31–35]. In this setting the incidence of thermal injury to the bowel has been noted to range from 1/360 to 1/7300 laparoscopic sterilization procedures [32,34–36]. This assumption was also consistent with the delayed presentation of patients with bowel injury. The clinical presentation of these seems to follow a sequence of bowel burn, followed by necrosis and delayed perforation.

The assumption that electricity is the most common cause of injury has been challenged. Levy and Soderstrom using an animal model have demonstrated the distinct histologic features of puncture wounds in comparison to electrical injury (Fig. 2) [37]. Veress and trocar puncture wounds were noted to have distinctive histologic features which included non-coagulative necrosis, capillary in growth, white cell infiltration, fibrin deposition and significant reconstruction of the injured muscle coat. By contrast bipolar and monopolar electrical injuries were noted to have extensive coagulative necrosis, with absent capillary ingrowth, inflammatory response, or fibroblastic reconstruction. These latter findings have been confirmed by others [38]. A Mallory trichrome stain confirms the presence of coagulative necrosis secondary to electrical injury by staining smooth muscle red and injured tissue and collagen blue [37]. A picrous red stain and polarized light may be a more specific method to confirm electrical injury to tissue [24].

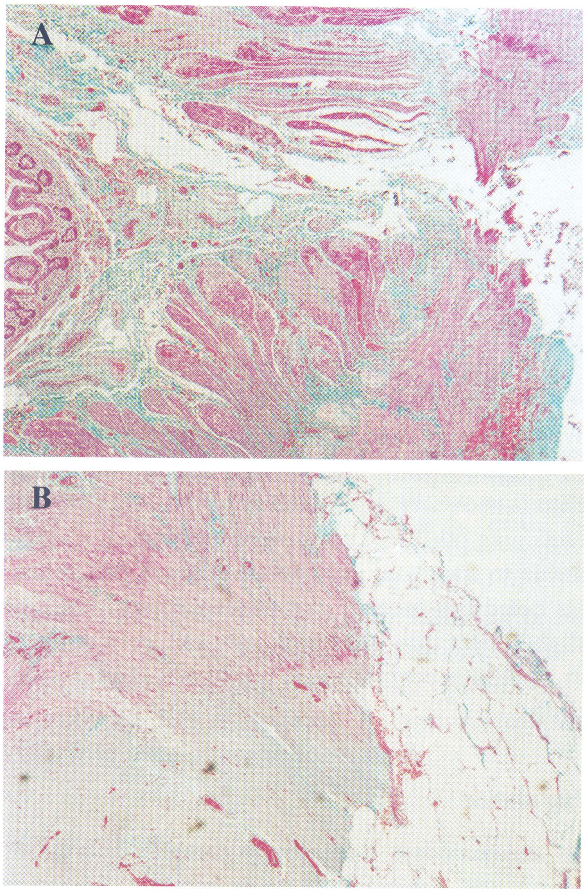

FIGURE 2 Histophotographic (low power) appearance of acute traumatic (A) and thermal (B) bowel injuries using a Mallory Trichrome stain. Note how the muscularis retains its pink color in the traumatic injury (A) but loses this stain in the burned bowel (lower half of (B)).

Ryder and Hulka have attempted to delineate the histologic extent of tissue damage using bipolar cautery [38]. Using a porcine model they applied electrical energy via Kleppinger forceps to tissue at predetermined distances from bowel. They demonstrated that even at 1 cm from the bowel, electric coagulation resulted in an acute inflammatory response and tissue edema limited to the serosa. When coagulation was applied immediately adjacent to the bowel wall, the inflammatory response extended to muscular layer of the bowel wall and into the surrounding submucosa. In all cases the mucosa was histologically intact, unless the full thickness of the bowel had been coagulated.

Soderstrom reported on 66 litigated cases referred for review because of unrecognized bowel injuries. While 89% of injuries were presumed to represent bowel burns, only 9% of cases met the histologic criteria necessary to substantiate such injury [24]. The remaining 60 (91%) cases were histologically attributable to traumatic intraoperative bowel perforation. He noted that patients with thermal injury presented slightly later than those with traumatic perforation; 5 to 15 days in comparison to one to nine days, respectively.

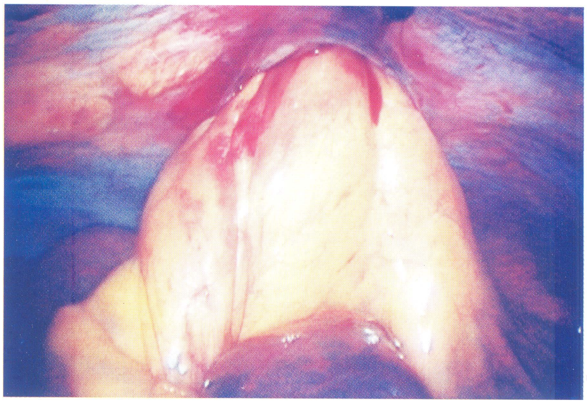

FIGURE 3A Small bowel herniation through a left lower abdominal port site as viewed from the umbilicus. Note that part of the mesentery of the small bowel has herniated.

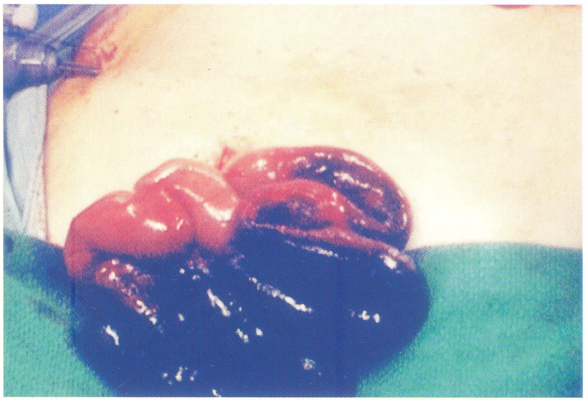

FIGURE 3B Loops of herniated small bowel were exteriorized through a minilaparotomy over the previous port site.

### Herniation

To accommodate increasingly complex instrumentation and procedures, many modern operative laparoscopic procedures require an increase in the number and size of ports. With more and larger defects in the anterior abdominal wall, there is an increased risk for herniation.

The incidence of bowel herniation following laparoscopic surgery recently has been estimated to range between 0.02 and 1% [2–4]. There are currently 35 reported cases of laparoscopic surgery complicated by bowel herniation, from which useful insights can be gleaned [2–9]. Port size is an important prognostic factor for this complication. Forty-one to 66% of such events occur with port sizes greater than or equal to 12 mm, while 33% to 45% occur with a 10 mm port size [3,4]. Except for an omental herniation through a 5 mm fascial defect, no other herniations have been reported with smaller port sizes [10]. The location of ports is also an important risk factor. To date a preponderance of herniations have occurred at extra-umbilical sites (76%) as compared to the umbilicus (24%) [4]. Most commonly small bowel is involved (86%) (Fig. 3), although herniations involving the omentum, cecum and ascending colon have been reported [4]. Six cases of Richters hernia (partial incarcerations of a portion of the circumference of the bowel) have been reported [11–15]. Fascial screws, extirpative procedures and extensive manipulation of the laparoscopic ports are associated with larger fascial defects, and have been identified as potential risk factors for this complication.

The etiology of bowel herniation is multifactorial. In some instances herniation occurs during the

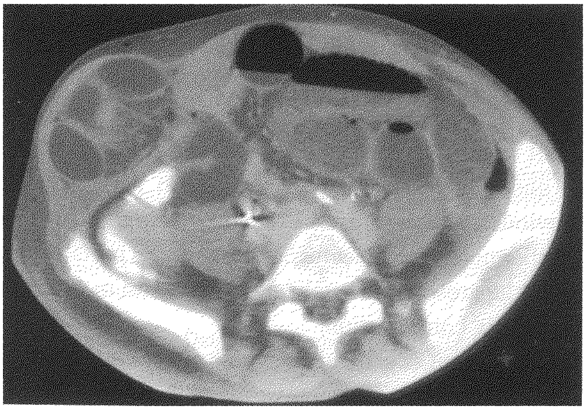

FIGURE 4 Abdominal CT scan illustrates small bowel loops above fascia in a patient with a herniation through a 12 mm right lateral trocar site.

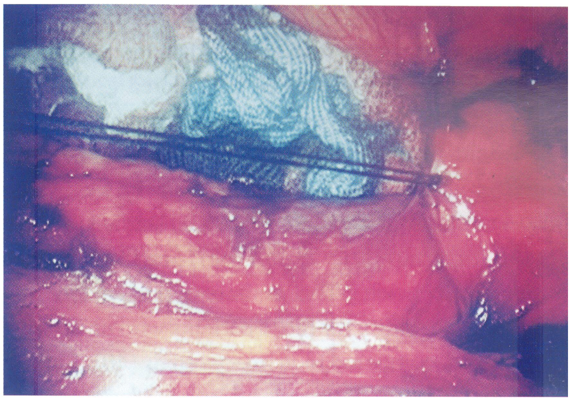

FIGURE 5 Superficial thermal injury to the serosa of the transverse duodenum repaired by laparoscopically placing imbricating silk stitches to correct the defect. The injury occurred while removing the lymph nodes overlying the vena cava (shown in the center of the photo sandwiched between the aorta and a minilaparotomy pad).

removal of sleeves, or with involuntary Valsalva upon awakening from anesthesia. In all cases, a fascial defect exists which is large enough to accommodate bowel. Closure of the fascia through a small skin incision can be difficult with traditional techniques. It is not surprising then that hernias can occur despite reports of attempted closure in as many as 17% to 50% of cases [2–4]. In many more instances fascial closure was not attempted.

The clinical presentation of bowel herniations can be quite varied. Complaints can be non-specific and include abdominal pain, distension, mass, nausea and vomiting. According to existing literature, the diagnosis is made clinically in 32% of cases, by computed tomography in 42% (Fig. 4), via conventional radiography 16%, and at surgery in 11% of cases [4]. Patients with this complication usually present after the eighth postoperative day, although herniations have been recognized as early as the day of surgery or as late as one year post surgery.

Management of laparoscopic incisional hernias (from the previously mentioned reference) has involved reoperation, usually open laparotomy or minilaparotomy, with bowel resection required in 29% of cases. Herniations have been managed laparoscopically. Successfully executed, this approach can potentially reduce the morbidity and length of hospital stay, while maximizing cosmesis. Even cases requiring bowel resection may be approached laparoscopically without compromising patient care. Close observation and follow up may be appropriate for otherwise asymptomatic herniations.

### Management

The successful management of initially unrecognized bowel injuries varies. Significant thermal damage or lacerations often necessitate resection of the involved bowel segment. Small bowel injuries can be oversewn, excised or resected (Fig. 5). Large bowel injury usually requires colostomy [36].

In contrast, the bowel injury that is recognized at the time of laparoscopy offers more flexibility in the management. Whether thermal or traumatic, small or large bowel, injuries may be managed either laparoscopically or via laparotomy. Laparoscopic repairs can be accomplished using endoscopic stapling or suturing techniques, as well as copious irrigation [16–18,39]. In the face of a large amount of fecal contamination and or extensive bowel injury, open laparotomy should be considered even in the hands of the expert laparoscopist. It is important to recognize that thermal injuries can produce damage which extends beyond the visual limits of the injury.

A simple purse string can be placed around superficial bowel burns, however with greater damage a resection with 4 cm margins should be considered [29].

Recent research suggests that recognize large bowel injuries can be repaired primarily without the need for colostomy, even with unprepared bowel. One prospective randomized controlled trial studied primary closure of traumatic colon perforations versus temporary diversion. The authors report a ten fold increase in morbidity for those patients who underwent colostomy as compared to primary repair [40]. Similar results have been obtained by other investigators. Ultimately the choice of approach to the repair depends on the preference and experience of the individual surgeon.

### Recommendations

Adequate fascial closure remains the cornerstone of prevention for bowel herniations following laparoscopy. In most instances fascial defect repair is either not attempted, or inadequately accomplished. Placement of fascial closure stitches under direct laparoscopic visualization should reduce the incidence of this complication. New devices and laparoscopic techniques to facilitate this procedure have been described by other authors [41,43,44].

Likewise the avoidance of large ports and fascial screws, especially outside of the midline, can be important in reducing the likelihood of this complication. Decompression of the pneumoperitoneum, and the removal of all trocar sleeves under laparoscopic visualization and with open port valves can be expected to reduce the likelihood of herniation associated with these procedures. Finally, the closure of the fascial wall defect repair prior to the reversal of anesthetic relaxant is important in reducing the likelihood of Valsalva which can also be associated with the development of hernias.

Traumatic and thermal bowel injury can also be prevented and its management facilitated by adequate skill and planning. A thorough preoperative mechanical bowel preparation and routine orogastric tube placement prior to trocar placement, decreases the likelihood of bowel injury and facilitates conservative management should it occur. Likewise primary access to the abdominal cavity away from the site of potential adhesions, is important in avoiding bowel injury during entry and subsequent adhesiolysis. The use of a left upper quadrant primary access can avoid injury to underlying structures, especially in patients with previous midline incisions or umbilical hernias [45]. Furthermore, the placement of ancillary ports under direct laparoscopic visualization is crucial in order to avoid this complication.

Likewise, it is critical that the healthcare providers likely to triage post-operative patients, such as emergency room physicians and residents, be educated regarding the subtle clinical presentation which typically occurs with unrecognized bowel injury. A high index of suspicion should be maintained for all post operative laparoscopy patients who present even with mild non-specific complaints. Patients who undergo operative laparoscopic procedures, and who are not improving require careful evaluation and observation. When confronted with a patient who is not improving, the surgeon should move expeditiously to exploratory laparoscopy or laparotomy. Procrastination can prove fatal.

### CONCLUSION

Modern operative laparoscopy has extended the range and complexity of procedures which can be performed by gynecologists. Bowel injury is an inherent risk of operative laparoscopy and represents perhaps the most important complication of this new technology. With adequate training and education, injury can be prevented or recognized early and appropriately managed. The net effect will be to substantially reduce morbidity and mortality, and increase the accessibility of this new technology to an ever increasing number of women.

### *References*

[1] Hulka, J.F., Peterson, B., Phillips, J.M. and Surrey, M.W. Operative laparoscopy: American Association of Gynecologic Laparoscopists 1991 membership survey. *J. Reprod. Med.* 1993; **38**: 569–571.

[2] Kadar, N., Reich, H., Liu, C.Y. *et al.* Incisional hernias after major laparoscopic gynecologic procedures. *Am. J. Obstet. Gynecol.* 1993; **168**: 1493–1495.
[3] Montz, F.J., Holschneider, C.H. and Munro, M.G. Incisional hernia following laparoscopy: A survey of the American Association of Gynecologic Laparoscopists. *Obstet. Gynecol.* 1994; **84**: 881–884.
[4] Boike, G.M., Miller, C.E., Spirtos, N.M. *et al.* Incisional bowel herniations after operative laparoscopy: A series of nineteen cases and review of the literature. *Am. J. Obstet. Gynecol.* 1995; **172**: 1726–1733.
[5] Schiff, I. and Naftolin, F. Small bowel incarceration after uncomplicated laparoscopy. *Obstet. Gynecol.* 1974; **43**: 674–675.
[6] Kurtz, B.R., Daniell, J.F. and Spaw, A.T. Incarcerated incisional hernia: A case report. *J. Reprod. Med.* 1993; **38**: 643–644.
[7] Plaus, W.J. Laparoscopic trocar site hernias. *J. Laparoendosc. Surg.* 1993; **3**: 567–570.
[8] Storms, P., Stuyven, G., Vanhemelen, G. and Sebrechts R. Incarcerated trocar wound hernia after hysterectomy. *Surg. Endosc.* 1994; **8**: 901–902.
[9] Jones, D.B., Callery, M.P. and Soper, N.J. Strangulated incisional hernia at trocar site. *Surg. Laparoscopic Endosc.* 1996; **6**: 152–154.
[10] Toub, D.B. and Campion, M.J. Omental herniation through a 5-mm laparoscopic cannula site. *J. Am. Assoc. Gynecol. Laparosc.* 1994; **1**: 413–414.
[11] Bourke, J.B. Small-intestinal obstruction from a Richter's hernia at the site of insertion of a laparoscope. *Br. Med. J.*, 1977; **2**: 1393–1394.
[12] Kiilholma, P. and Makinen, J. Incarcerated Richter's hernia after laparoscopy: A case report. *Eur. J. Obstet. Gynecol. Reprod. Biol.* 1988; **28**: 75–77.
[13] Hill, D.J., Maher, P.J., Wood, C.E. *et al.* Complications of laparoscopic hysterectomy. *J. Am. Assoc. Gynecol. Laparosc.* 1994; **1**: 159–162.
[14] Wasserman, S.A. Incarcerated hernia after laparoscopically assisted vaginal hysterectomy. *J. Am. Assoc. Gynecol. Laparosc.* 1994; **1**: 415–416.
[15] Williams, M.D., Flowers, S.S., Fenoglio, M.E., *et al.* Richter hernia: A rare complication of laparoscopy. *Surg. Laparosc. Endosc.* 1995; **5**: 419–421.
[16] Reich, H., McGlynn, F. and Budin, R. Laparoscopic repair of full-thickness bowel injury. *J. Laparoendosc. Surg.* 1991; **1**: 119–122.
[17] Reich, H.R. Laparoscopic bowel injury. *Surg. Laparosc. Endosc.* 1992; **2**: 74–78.
[18] Nezhat, C., Nezhat, F., Ambroze, W., *et al.* Laparoscopic repair of small bowel and colon: A report of 26 cases. *Surg. Endosc.* 1993; **7**: 88–89.
[19] Penfield, A.J. How to prevent complications of open laparoscopy. *J. Reprod. Med.* 1985; **30**: 660–663.
[20] Yuzpe, A.A. Pneumoperitoneum needle and trocar injuries in laparoscopy: A survey on possible contributing factors and prevention. *J. Reprod. Med.* 1990; **35**: 485–490.
[21] Kaali, S.G. and Barad, D.H. Incidence of bowel injury due to dense adhesions at the sight of direct trocar insertion. *J. Reprod. Med.* 1992; **37**: 617–618.
[22] Lehman, E., Riedel, H., Mecke, H. and Semm, R. Pelviscopy/Laparoscopy and its complications in Germany 1949–1988. *J. Reprod. Med.* 1992; **37**: 671–673.
[23] Crist, D.W. and Gadacz, T.R. Complications of laparoscopic surgery. *Surg. Clin. North Am.* 1993; **73**: 265–288.
[24] Soderstrom, R.M. Bowel injury litigation after laparoscopy. *J. Am. Assoc. Gynecol. Laparosc.* 1993; **1**: 74–77.
[25] Khare, V.K., Martin, D.C. and Kaigh, J. Buttonhole ulceration and perforation of the rectum. *J. Am. Assoc. Gynecol. Laparosc.* 1995; **1**: 12–15.
[26] Tsai, F.P., Chen, P.J., Lee, C.L. *et al.* Major complications during laparoscopy assisted vaginal hysterectomy: Report of four cases and review of the literature. *Chang Gung Med. J.* 1995; **18**: 52–57.
[27] Gentile, G.P. and Siegler, A.M. Inadvertent intestinal biopsy during laparoscopy. *Fertil. Steril.* 1981; **36**: 402–404.
[28] Meeks, G.R., Meydtrech, E.F., Waller, G.A. *et al.* Unscheduled hospital admission following ambulatory gynecologic procedures. *J. Gynecol. Surg.* 1993; **9**: 227–233.
[29] Soderstrom, R.M. and Levy B.S. Bowel injuries during laparoscopy: Causes and medico-legal questions. *Contemp. Obstet.* 1986: 41–45.
[30] Davis, G.D., Wolgmott, G. and Moon, J. Laparoscopically assisted vaginal hysterectomy as definitive therapy for stage III and IV endometriosis. *J. Reprod. Med.* 1993; **38**: 577–581.
[31] Peterson, H.B., Ory, H.W., Greenspan, J.R. *et al.* Deaths associated with laparoscopic sterilization by unipolar electrocoagulation devices 1978 and 1979.*Am. J. Obstet. Gynecol.* 1981; **139**: 141–143.
[32] Jordan, J.A., Edwards, R.L., Pearson, J. and Maskery, P.J.K. Laparoscopic sterilization and follow-up hysterosalpingogram. *J. Obstet. and Gynaecol. Br. Cmnwlth.* 1971; **78**: 460–466.
[33] Saltzein, E.C., Schwartz, S.F. and Levinson, C.J. Perforation of the small intestine secondary to laparoscopic tubal cauterization. *Ann. Surg.* 1973; **178**: 34–36.
[34] Thompson, B.H. and Wheeless, C.R. Gastrointestinal complications of laparoscopy sterilization. *Obstet. Gynecol.* 1973; **41**: 669–676.
[35] Maudsley, R.F. and Qizilbash, A.H. Thermal injury to the bowel as a complication of laparoscopic sterilization. *Can. J. Surg.* 1979; **22**: 232–234.
[36] Chi, I. and Kennedy, K.I. Early readmission following elective laparoscopic sterilization: A brief analysis of a rare event. *An. J. Obstet. Gynecol.* 1984; **148**: 322–327.
[37] Levy, B.S., Soderstrom, R.M. and Dail, D.H. Bowel injuries during laparoscopy: Gross anatomy and histology. *J. Reprod. Med.* 1985; **30**: 168–172.
[38] Ryder, R.M. and Hulka, J.F. Bladder and bowel injury after electrodesiccation with Kleppinger bipolar forceps: A clinicopathologic study. *J. Reprod. Med.* 1993; **38**: 595–598.
[39] Leuken, R.P., Hesse, U. and Kuchlin, J. Bowel injury at laparoscopic adhesiolysis – An absolute indication for laparotomy? *Gynaecol. Endosc.* 1992; **1**: 39–42.
[40] George, S.M., Fabian, T.C., Voeller, G.R. *et al.* Primary repair of colon wounds: A prospective trial in non-selected patients. *Ann. Surg.* 1989; **209**: 728–733.
[41] Carter, J.E. A new technique of fascial closure for laparoscopic incisions. *J. Laparoendosc. Surg.* 1994; **4**: 143–148.
[42] Jager, R.M. Safe repair of umbilical fascial wounds. *J. Laparoendosc. Surg.* 1994; **4**: 199–200.
[43] Goldrath, M. and Phillips, E. A method for closing laparoscopy port incisions using a modified Veress needle. *J. Am. Assoc. Gynecol. Laparosc.* 1995; **3**: 287–290.
[44] Pelosi, M.A. The Pelosi technique of full-thickness cannula site closure. *J. Am. Assoc. Gynecol. Laparosc.* 1995; **3**: 291–293.
[45] Childers, J.M., Brzechffa, P.R. and Surwit, E.A. Laparoscopy using the left upper quadrant as the primary trocar site. *Gynecol. Oncol.* 1993; **50**: 221–225.

